# Stress Management Among Caregivers of Detained Youth: Protocol for Randomized Controlled Trial of the RAISE Web-Based mHealth App

**DOI:** 10.2196/67511

**Published:** 2025-07-10

**Authors:** Johanna B Folk, Adrian Aguilera, Tara M Chaplin, Marina Tolou-Shams

**Affiliations:** 1 Department of Psychiatry and Behavioral Sciences School of Medicine University of California, San Francisco San Francisco, CA United States; 2 School of Social Welfare University of California, Berkeley Berkeley, CA United States; 3 Department of Psychology George Mason University Fairfax, VA United States

**Keywords:** adolescence, community engagement, digital health technology, incarceration, mindfulness, randomized clinical trial

## Abstract

**Background:**

Detained adolescents exhibit high rates of behavioral health needs, yet few receive treatment during detention or community re-entry. Once adolescents are released into the community, caregivers must mobilize significant resources and overcome barriers to facilitate their treatment engagement. Parenting stress is often heightened during this forced separation and the re-entry transition. Parenting stress is associated with greater perceived barriers to treatment and, for adolescents who begin treatment, less therapeutic change and premature treatment dropout. Interventions designed to support caregivers of detained adolescents in managing their stress while navigating the juvenile legal system are urgently needed, and mobile health (mHealth) interventions offer promising, scalable approaches. RAISE (Reducing pArentIng StrEss) is a web-based application co-designed with caregivers of detained adolescents to reduce caregiver stress and promote postrelease adolescent behavioral health services use.

**Objective:**

This study will evaluate the effectiveness of RAISE in reducing caregiver stress and promoting adolescent behavioral health services use following release from detention.

**Methods:**

A randomized controlled trial with 60 caregivers of detained adolescents (ages 12-17 years) across the United States will be conducted. Caregivers will be recruited through passive and active techniques and randomized to receive RAISE (intervention) or an informational brochure (comparison). Self-assessment questionnaires will be completed at baseline and 3- and 6-month follow-up timepoints. The fully automated RAISE intervention includes an 8-week stress reduction intervention, self-monitoring and affirmational SMS text messaging, and resources related to navigating the juvenile legal system. Assessments include empirically validated measures of parenting stress, mindful parenting, parenting self-efficacy, adolescent services use, motivation for youth treatment, caregiver behavioral health, sociodemographics, and RAISE usability (intervention only). Caregivers will also participate in a semistructured qualitative exit interview at the 3-month (postintervention) timepoint. Descriptive statistics will examine recruitment, randomization, assessment, retention, and application usability. Independent samples *t* tests and chi-square analyses will determine whether randomization was successful based on multiple background variables; group differences will be accounted for in outcome analyses. Regression analyses will be used for outcome analyses, with an intent-to-treat design; analyses will include intervention group as a predictor and control for the baseline level of the outcome, application usage, and demographic characteristics. Potential moderators and mediators of intervention effects will be explored.

**Results:**

We propose the enrollment of 60 caregivers by April 2025, final data collection by September 2025, and submission of main findings for publication in December 2025.

**Conclusions:**

This study will provide empirical evidence regarding the impact of an mHealth stress reduction intervention co-designed with caregivers of detained adolescents. Findings will be informative for legal systems regarding how best to support caregivers of detained adolescents and the impact of reducing caregiver stress on adolescents’ linkage to behavioral health services following their release into the community.

**Trial Registration:**

ClinicalTrials.gov NCT05032742; https://clinicaltrials.gov/study/NCT05032742

**International Registered Report Identifier (IRRID):**

DERR1-10.2196/67511

## Introduction

### Background

Although rates of youth incarceration have declined over the past decade, the most recent publicly available data show that in 2021, the United States incarcerated over 24,000 young people [1]. This relatively low number, compared to historical rates (eg, in 2019, there were over 35,000 youth detained), is in part due to the COVID-19 pandemic, which had significant impacts on juvenile legal system facilities and reporting of data related to youth in placement [1].

Detained adolescents are disproportionately male, from racially minoritized backgrounds, and most commonly incarcerated for delinquent (violent, property, etc) rather than status (ie, behaviors criminalized for minors but not adults) offenses [2]. About two-thirds of detained males and three-quarters of detained females have a diagnosable psychiatric condition [3], with nearly half meeting criteria for a substance use disorder. Many have co-occurring substance use and psychiatric disorders, increasing their risk for substance-related recidivism [4], persistent reoffending [5], and antisocial activity [6].

Behavioral health treatment is effective at improving adolescent behavioral health (eg, reducing substance use and improving mental health) and legal outcomes, with research consistently demonstrating that treatment involving adolescents and their caregivers is most efficacious in offsetting maladaptive trajectories of risk behavior [7,8]. Yet, few adolescents with behavioral health needs receive services or referrals during detention or community re-entry [9], and legally involved adolescents and their caregivers face multiple barriers to accessing community-based treatment. Adolescent behavioral health treatment access and engagement are multifaceted issues. At the individual level, adolescents and caregivers may experience stigma about accessing behavioral health services, not trust service providers, or may not feel services are needed [10,11]. At the structural level, factors such as lack of available providers, transportation, and insurance access can also impact access [10]. Ultimately, caregivers must often mobilize significant resources to facilitate adolescent engagement in community-based behavioral health treatment (transportation, childcare, etc); this can be challenging when juggling the stress of caring for a legally involved adolescent. As such, few legally involved adolescents in need of treatment actually receive any of the empirically based services available [9]. Detention is a critical window in which adolescents and their caregivers can be linked with treatment, yet as a nation, we are largely failing to use this opportunity.

### Parenting Stress

Among the potentially malleable factors contributing to adolescent treatment engagement is parenting stress (ie, stress reaction to demands of parenting), a well-documented impediment to treatment engagement, treatment progress, and a predictor of adverse youth outcomes such as substance misuse. Parent-child relationship (P-C-R) stress theory [12,13], one of the most widely tested and empirically supported parenting stress theories, posits that parenting stress arises from 3 domains: (1) parent (P); (2) child’s behavior (C); and (3) parent-child relationship (R). Each domain is associated with functional outcomes (eg, P with parent psychopathology [14,15], C with child behavior problems and psychopathology [16,17], R with parent-child relationship conflict [18,19]), which degrade the quality and effectiveness of parenting behavior and lead to increases in child emotional and behavioral problems. P-C-R theory proposes bidirectional effects (eg, child’s behavioral difficulty increases over time and parenting stress increases, resulting in further problems with parenting and child well-being; parent mental health leads to impaired parenting and increases child behavioral problems, which increases parenting stress). Caregivers’ reactions to the stress of parenting are key in propelling the process forward. For caregivers of legally involved adolescents, stress can be amplified by separation from their detained child and navigating the expectations imposed by the carceral system.

Parenting stress is associated with greater perceived barriers to treatment (eg, perceived relevance) [20], less therapeutic change among youth during treatment [21], and premature treatment dropout [20,22,23]. Highly stressed parents tend to report less parenting self-efficacy [24] and more negative parenting practices [25] that increase youth risk for substance misuse [26]. Higher parental stress has been associated with greater motivation for youth treatment (ie, recognition that the youth has a problem and caregiver readiness to participate in their treatment), particularly when caregivers perceive youth to have more severe symptoms [27]. Among caregivers of legally involved adolescents in the community, caregivers experiencing more mental health distress tend to be more likely to recognize when their child is experiencing a behavioral health problem [28]; however, for caregivers of adolescents who are mandated to treatment by the courts, recognition of youth behavioral health problems has been shown to be lower compared to those referred by other sources (self, school, etc) [27]. Caregivers of adolescents re-entering the community following detention face a myriad of stressors and demands, for example, ensuring their youth adheres to probation requirements (eg, can include enrollment in school, court-mandated community service, and court-mandated service participation). Among the range of problems that adolescents re-entering the community are facing, caregivers may be less likely to recognize the need for behavioral health treatment as a priority. Disrupting the parent-child relationship cycle by reducing parenting stress has the potential to increase caregivers’ self-efficacy to engage in effective parenting strategies needed to overcome barriers in facilitating adolescent treatment engagement and to recognize when their youth needs behavioral health intervention.

**Figure 1:**
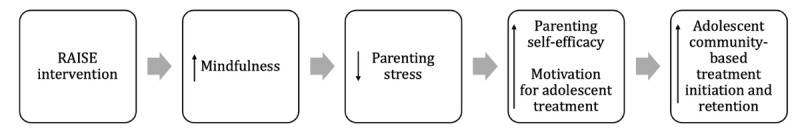
Hypothesized model of intervention effects.

One promising method of reducing parenting stress is through mindfulness-based interventions [29]. Mindfulness, “the practice of focusing full attention on the present moment intentionally and without judgment” [30], is posited to reduce perceived and biological stress reactivity by increasing awareness of and ability to tolerate thoughts and emotions. Evidence suggests that in-person mindfulness-based parenting interventions increase mindfulness, decrease parenting stress [31,32], and improve parent-adolescent relationship quality among highly stressed parents [33]. Decreasing parenting stress, starting while adolescents are in detention, has the potential to enhance caregivers’ ability to facilitate adolescent engagement in community-based behavioral health treatment during re-entry; possible mechanisms of action (Figure 1) include increases in caregivers’ parenting self-efficacy (ie, beliefs about one’s ability to carry out parenting roles [34]) and motivation for adolescent-focused treatment.

### mHealth Technology Approaches to Intervention

Parenting stress is a malleable individual-level target that can be addressed using approaches that circumvent barriers to treatment access, such as digital mobile health (mHealth) technology. Research with postpartum mothers experiencing parenting stress and parents of primary school students provides initial support for the effectiveness of self-guided, web-based mindfulness and self-compassion training programs [35,36]. Increasing available providers, reducing the cost of services, and improving reliable transportation access can also enhance treatment accessibility but require intensive, long-term system and policy-level interventions.

Using mobile phones to deliver health (mHealth) interventions is a viable method for caregivers of legally involved adolescents, who commonly cite time, transportation, agency or program issues, and distrust of professionals as barriers to accessing in-person services [10]. mHealth technology refers to “the use of mobile devices, such as mobile phones, tablet computers, and personal digital assistants to provide medicine, public health, and health services” [37] and is an efficacious, low-cost way of reaching underserved populations to engage them in and deliver quality care [38]. mHealth circumvents many barriers caregivers of legally involved adolescents report, including transportation and time (eg, accessible from any internet-connected device at one’s convenience). mHealth is widely used and effective at increasing communication, monitoring, and education, to reduce the burden of diseases associated with poverty, to improve access to health services, clinical diagnosis, and treatment adherence, and for chronic disease management [39,40]. mHealth allows for instantaneous and portable access and, in some cases, direct communication with providers [41]. Research indicates caregivers of legally involved adolescents and juvenile legal system stakeholders are interested in mHealth treatment [2,42,43], making it a likely acceptable approach for them. Using mHealth apps with caregivers of legally involved adolescents is, therefore, one potentially efficacious way to reduce parenting stress, circumvent well-documented barriers to treatment, and ultimately increase adolescent engagement in community-based behavioral health treatment.

### Participatory Co-Design Approaches to mHealth Intervention Development

Caregivers of detained adolescents have unique factors that require consideration when designing interventions and should be involved in the development process. Of note, caregivers often feel blamed for their child’s involvement in the system, internalize the stigma of being a “bad parent” [44], and feel overwhelmed by not understanding the court system and concerns for their child [44]. Further, they often harbor mistrust in the legal system, in part stemming from systemic racism [45] and a history of difficulty accessing effective community services and supports [46]. They are typically not included in or consulted regarding the development of interventions to help them, and as such, many existing interventions are not feasible or acceptable to them. Efforts to create relevant and accessible interventions must include their direct perspectives and buy-in to be successful.

Based upon principles of Community Partnered Participatory Research [47] and user-centered design [48,49], participatory informatics [50] is an approach to technology co-development that does not require technological expertise and includes users as equitable members in the design process. Involving caregivers in the design process increases the chances the mHealth intervention will be suitable for their needs, more satisfactory [49], and ultimately more effective at facilitating youth engagement in behavioral health treatment post release (via caregiver-level mechanisms of action; Figure 1).

### Study Objectives

This study will be the first to evaluate a mHealth intervention (RAISE app), designed through participatory co-design approaches with caregivers of detained adolescents, specifically focused on supporting caregivers of detained adolescents in managing their own stress while navigating the juvenile legal system. Using a randomized controlled trial (RCT) design, the primary goal of the study is to assess the feasibility, acceptability, and preliminary impact of the RAISE intervention on proximal caregiver outcomes (eg, parenting stress, parenting self-efficacy, and motivation for youth treatment) and more distal adolescent community-based treatment engagement outcomes.

#### RAISE App

The RAISE app was co-designed using participatory informatics approaches and the secure, web-based Chorus platform (Chorus Innovations, Inc) [51]. The Chorus platform provides a simple, visual, web interface to create interactive mHealth apps without requiring programming skills. Chorus includes interactive voice, texting, and mobile-based web app capabilities and is accessible from any device with internet browsing capabilities.

The co-design process is described in detail elsewhere (Folk et al, unpublished data). In brief, 6 caregivers of adolescents (12-18 years old) who had been detained in juvenile hall within the past year (including 50% currently detained) participated in 9 co-design sessions. Caregivers met either individually (n=2) or in pairs (n=4) with members of the research team to provide in-depth input regarding the needs of caregivers with detained adolescents, perspectives on acceptable intervention approaches (including reviewing mindfulness-based stress reduction approaches), and to develop and refine all RAISE app content. Once an initial version of the RAISE app was developed, co-designers and 6 new caregivers tested the app over a 3-month period and participated in 3 feedback sessions. The app was then refined in preparation for the current pilot trial.

**Figure 2:**
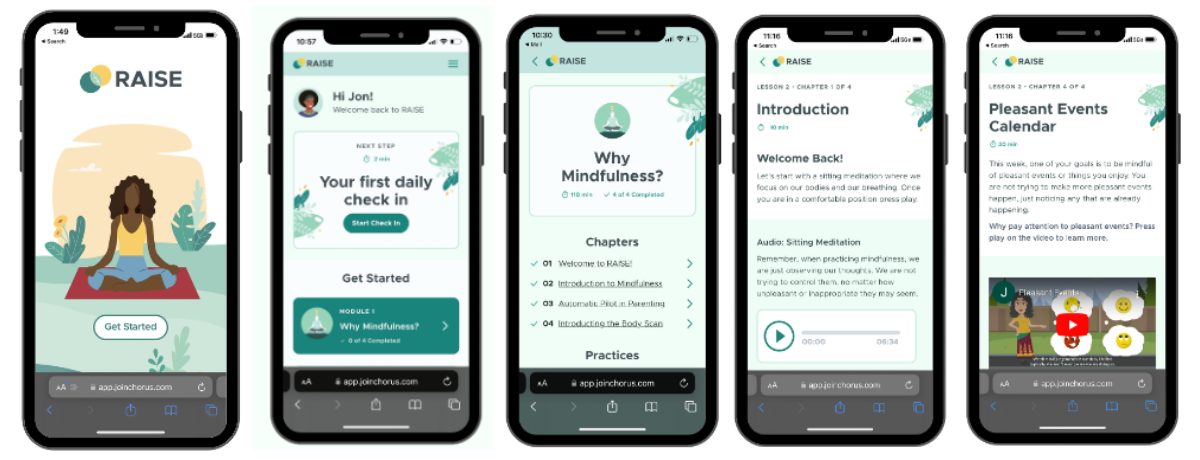
Select pages of RAISE application.

The web-based RAISE app contains: (1) an 8-week stress-reduction intervention adapted from a mindful parenting curriculum [33]; (2) self-monitoring SMS text messages related to stress, mindfulness, and parenting self-efficacy (daily for the first 8 weeks, then reducing to 3 times per week for 3 weeks and weekly for the final 12 weeks); (3) weekly motivational SMS text messages, written by co-designers; and (4) resources related to navigating the juvenile legal system and accessing behavioral health treatment. Example pages within the RAISE app are displayed in Figure 2. The RAISE intervention is entirely self-guided, and the app can be accessed as frequently as a caregiver chooses; new content is released weekly for the 8-week stress-reduction intervention, with the option to revisit prior weeks’ material at any time. Each module contains mixed-media approaches to instruction, including audio-recorded exercises, videos (animated, with the exception of a yoga instruction video filmed by a live person), images, written text, and writing prompts to reflect on the content. The app will be “frozen” to changes during the trial (with the exception of addressing any unanticipated bugs), so all participants receive the same version of the intervention. During the informed consent appointment, users randomized to RAISE will set up an account with the assistance of a research assistant and be shown a tutorial video with key application features (which is also available in the app for their viewing at any time); no additional training is provided, however, if users experience technical difficulties with the application, they can contact the research team for support.

#### Hypotheses

We hypothesize that caregivers receiving the RAISE intervention will experience reductions in their parenting stress and that their adolescents will be more likely to initiate behavioral health services post release. We will explore hypothesized mechanisms of intervention impact (eg, mindful parenting, parenting self-efficacy, and motivation for youth treatment) as well as potential moderators (eg, gender, race, and ethnicity).

## Methods

### Study Design

**Figure 3:**
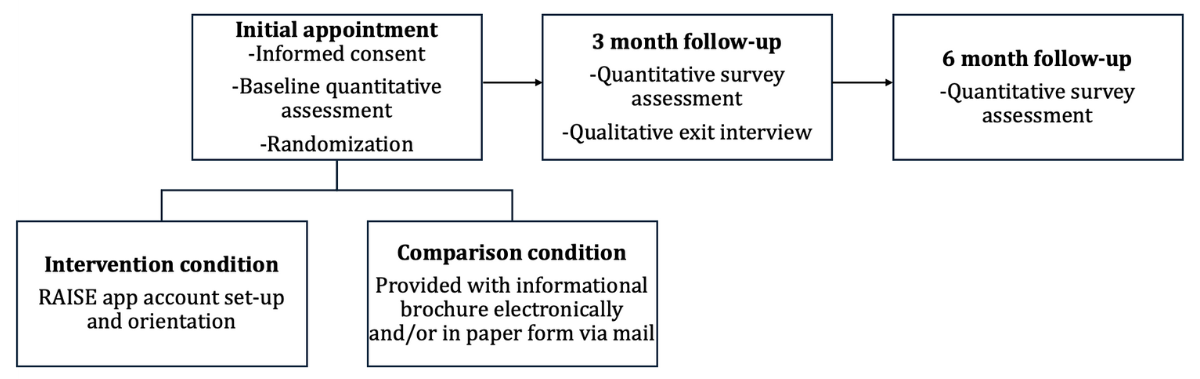
Study flow diagram.

Caregivers of detained adolescents (N=60) will participate in the RCT to assess the preliminary impact of the RAISE intervention on caregiver stress and adolescent behavioral health services use post release. A study flow diagram is displayed in Figure 3. Caregivers of adolescents (12-17 years old) in placements mandated by the juvenile legal system will be enrolled in the study and randomized to either the RAISE intervention or to receive an informational brochure (comparison). Enrolled caregivers will complete a computerized set of questionnaires (on a tablet device, mobile phone, PC, or laptop computer) at baseline and 3- and 6-month follow-ups. The trial has been registered with ClinicalTrials.gov (NCT05032742) (Multimedia Appendices 1 and 2).

### Study Sample Size Determination

The main overall study focus is on the feasibility and acceptability of the codevelopment process and RAISE intervention; however, we will examine the direction of effects through this pilot RCT and cautiously consider effect size to inform sample size determination for a subsequent phase 2 efficacy trial. As with most pilot studies, these analyses are underpowered (for primary analyses considering a sample of 60 participants with α=.05, 4 of predictors, and estimating a medium effect size (Cohen f^2^=.15), power=.62). There are notable concerns with calculating effect sizes based upon pilot studies, including potential for overestimating and underestimating effects [52]. Given that we anticipate small to medium effect sizes, we followed published guidance to determine a planned enrollment of 30 participants per study arm [53].

### Intervention Condition (RAISE)

Caregivers (n=30) who are randomized to the RAISE intervention will receive access to the web application for a 6-month period. The first 3 months are considered the main intervention window, as this is the timeframe when the 8-week stress reduction intervention content is intended to be completed. Caregivers have the option to revisit this material at any time during the 6-month access period, and use of all RAISE app features is captured in app logs. Caregivers can access the web application on any device with an internet browser and log in using a unique username and password. RAISE is provided at no cost to participants.

### Informational Brochure Condition (Comparison)

Caregivers (n=30) who are randomized to the comparison condition will receive an informational brochure about the juvenile legal system. Content for the brochure was adapted from existing guides distributed to caregivers by local jurisdictions in partnership with juvenile legal system professionals [54].

### Recruitment

Enrollment for the trial commenced in October 2023 and is anticipated to conclude by April 2025. Caregivers are recruited through passive and active techniques. Flyers and business cards with a QR code, which when scanned directs to an online eligibility survey and video explaining the study, are posted in public places (detention center waiting areas, court buildings, etc) and distributed electronically by legal system partners (probation officers, attorneys, behavioral health clinicians, etc). Study staff also recruit caregivers through active means, specifically by talking directly with families during family visitation in detention centers. Caregivers who provide their contact information and permission to be contacted by the study team are offered an opportunity to learn more about the study by meeting with a research assistant either remotely via Zoom or in person. Caregivers who are eligible and interested in participating complete an informed consent discussion with a research assistant and the baseline assessment before randomization (Multimedia Appendix 3).

### Inclusion and Exclusion Criteria

Eligible caregivers must be the parent or legal guardian of an adolescent who (1) is currently detained by the juvenile legal system, (2) is 12-17 years old, (3) has an identified substance use or substance use and co-occurring mental health need, and (4) will ultimately be released into the community to the care of the enrolled caregiver. Computer or internet literacy is not an inclusion criterion for the study. Given that adolescents within this population are not always raised or cared for by their biological parents, we use the term “caregiver” to define the adult who has principal responsibility for the raising and care of the adolescent, and who can provide legal consent and enrollment in services and research studies. In addition, detention by the juvenile legal system can include secure facilities such as juvenile detention centers or alternative placements such as group homes, which are less secure, but where youth are not technically allowed to leave freely.

### Randomization

Participants will be randomized (using the REDCap [Vanderbilt University] randomization module [55,56]) to either the RAISE intervention group (30/60, 50%) or the informational brochure comparison group (30/60, 50%) directly after consent and completion of the baseline assessment. Randomization is blocked (to avoid “runs” of assignment to the same condition) and stratified by gender to ensure balance between the 2 conditions (because most caregivers are anticipated to identify as women). The randomization table is generated by a biostatistician and is not visible to the research assistant before assignment; no blinding was used. Participants will not be told which condition is the intervention of interest, but during the informed consent discussion are informed that they may be invited to test a mobile app.

### Ethical Considerations

This study was approved by the Institutional Review Board at the University of California, San Francisco (#20-32010) and certified by the Office for Human Research Protections. Caregivers will provide informed consent before completion of their baseline assessment during an appointment with a research assistant. Study data do not contain personally identifiable information and are coded with a unique study identification number; a list linking study identification numbers to specific participants is securely maintained and only accessible to trained study staff. Each participant will be offered a US $40 gift card as compensation for their time for each survey or interview completed (total possible compensation: US $160). A Data Safety and Monitoring Board was formed before the initiation of the RCT and includes 4 members with expertise in clinical trials, mental health research with underserved populations, and research with youth and families. Adverse events will be reported and managed in accordance with the institutional review board and the funder’s requirements.

#### Data Collection

Self-report data will be collected from caregivers through electronic quantitative surveys administered via REDCap (3 times total) and one-time qualitative interviews.

#### Quantitative Measures

##### Parenting Stress

The 90-item Stress Index for Parents of Adolescents [57] assesses caregivers’ perception of stressful areas in caregiver-adolescent interactions, including the relationship between stress and adolescent-caregiver characteristics, the quality of adolescent-caregiver interactions, and stressful life circumstances.

##### Mindful Parenting

The 8-item Interpersonal Mindfulness in Parenting Scale [58] assesses present-centered emotional awareness in parenting, present-centered attention in parenting, nonreactivity or low reactivity in parenting, and nonjudgmental acceptance in parenting.

##### Parenting Self-Efficacy

The 20-item Parenting Self-Efficacy Scale [59,60] assesses caregivers’ perceived parenting ability across 3 dimensions: parental connection, behavioral influence, and psychological autonomy.

##### Adolescent Services Use

The Child and Adolescent Services Assessment [61] obtains information about service use for mental health and substance use needs across multiple sectors (eg, schools and the legal system), including details about the type of treatment facility, professional discipline of provider(s), outpatient services, and out-of-home placements. We adapted this instrument to also assess whether services were mandated by the legal system. Outcomes of focus will include whether the adolescent completed any behavioral health services following release into the community (yes or no) and whether they complied with mandated services (yes or no). Barriers to adolescent services are also assessed (16 barriers; language, transportation, cost, stigma, etc). The assessment focuses on the 3 months before the adolescent’s detention (at baseline) and for follow-ups in the 3 months since the last assessment.

##### Motivation for Youth’s Treatment

The 8-item Motivation for Youth’s Treatment Scale [27] assesses intrinsic motivation for youth treatment through 2 subscales: (1) recognition that the youth has a problem and (2) readiness to participate in the youth’s treatment.

##### Caregiver Behavioral Health

The 23-item DSM-5 Level One Cross Cutting Symptom Inventory [62] assesses psychiatric symptoms (depression, anxiety, mania, psychosis, etc) and substance use during the past 2 weeks.

##### Acceptability of the RAISE App

For caregivers randomized to the RAISE condition only, the 18-item mHealth App Usability Questionnaire for Standalone mHealth Apps [63] assesses the acceptability (ie, ease of use, interface and satisfaction, and usefulness) of the RAISE app.

##### Sociodemographic Characteristics

We will collect data on caregiver race, ethnicity, gender, education, income, and adolescent history of legal involvement.

##### Application Use Metrics

We will extract use metrics from the Chorus platform. Metrics will include the number of days of use, number of modules completed, time spent practicing mindfulness exercises, and responses to self-monitoring messages sent by the application.

#### Qualitative Interviews with Caregivers

All caregivers (both conditions) will be invited to participate in a one-time qualitative interview at the time of the 3-month follow-up assessment. Interviews will focus on the feasibility, acceptability, and satisfaction with the RAISE app and informational brochure conditions (Multimedia Appendix 4).

#### Statistical Analysis

##### Quantitative Analysis

Analyses will be conducted using IBM SPSS Statistics software. Descriptive statistics will be used to examine issues related to recruitment (eg, sufficient numbers of caregivers consenting and pace of recruitment), randomization (eg, whether participants are willing to be randomized), assessment (eg, valid, reliable, acceptable, and complete), and retention (eg, rates of follow-up assessment completion). Preliminary analyses will examine whether the randomization process was successful, comparing groups on multiple background (gender, race, etc) and baseline (parenting stress, caregiver mental health, etc) variables through independent samples *t* tests (continuous variables) and chi-square analyses (categorical variables). If there are group differences, subsequent analyses will be run both with and without accounting for these variables. Efforts will be made at each stage of analysis to assess the importance of race and gender. Initially, means, distributions, and reliabilities for all major measures will be examined separately by race and gender. Any observed differences across demographic subgroups will be considered in the interpretation of substantive analyses. The proportion of missing data will be examined before outcome analyses to determine whether a complete case analysis is appropriate or whether multiple imputation is warranted (eg, if there is a large proportion of missing data, ie, >5%; if there are patterns in missingness based on demographic factors) [64].

#### RAISE User Engagement and Acceptability

RAISE app use metrics will be generated through logs from the Chorus platform. The use metrics will be examined in relation to the outcomes of interest (eg, changes in parenting stress) through bivariate correlations and linear regression and included as control variables when examining intervention effectiveness. Average response rates to daily ratings will be examined, as well as factors that relate to response rates (age, gender, race, mental health, substance use, etc) through bivariate correlations (eg, age) and independent samples *t* tests (eg, gender).

Descriptive statistics will be used to examine ratings of application usability (eg, ease of platform use) and acceptability of intervention components (content, mindfulness exercises, daily ratings, etc) on the mHealth App Usability Questionnaire for Standalone mHealth Apps [63].

#### RAISE Effectiveness

Outcome analyses will focus on proximal caregiver-level outcomes, including mindful parenting, parenting stress, parenting self-efficacy, perceived barriers to youth treatment, and motivation for youth treatment. Secondary distal outcomes will include adolescent treatment initiation and retention. Linear regression will be used for continuous outcomes (ie, mindful parenting, parenting stress, perceived barriers to youth treatment, motivation for youth treatment), logistic regression for 3- and 6-month follow-up dichotomous outcomes (ie, treatment initiation and adherence to mandated treatment), and Poisson or negative binomial regressions for count outcomes (ie, number of treatment sessions attended). All analyses will include the intervention group as a predictor and control for baseline level of the outcome variable, application usage, and demographic characteristics (determined during preliminary analysis). Potential moderators and mediators of intervention effects will be explored using the PROCESS macro in SPSS, which uses a regression framework. Moderation analyses will involve computing interaction terms of intervention group (intervention vs comparison) by key demographic factors (eg, caregiver age, gender, race, mental health, substance use, and youth age, centered before computing interaction term for continuous variables). Mediational effects will examine the impact of intervention group on key proximal outcomes assessed at 3 months (ie, mindful parenting, parenting stress, parenting self-efficacy, perceived barriers to youth treatment, and motivation for youth treatment) and their impact on distal youth treatment engagement and retention outcomes assessed at 6 months. An “Intent to Treat” design, where all initially assigned to the intervention group are included in analyses, whether or not they used the intervention, will be used.

##### Qualitative Analysis

Individual interviews will be digitally recorded and transcribed verbatim. Transcripts will be loaded into ATLAS.ti (ATLAS.ti Scientific Software Development), a qualitative analysis software for coding, text retrieval, intensive data management, and analysis. Interviews will be coded using inductive thematic analysis. An initial codebook will be developed based on the interview guides and corresponding transcripts. To improve reliability and ensure adequate intercoder agreement, the study principal investigator and research assistant will compare coding patterns and refine the codebook until consensus is reached, and then generate memos to highlight connections between codes and subcodes. Quotations will be compiled, and concepts and relationships pertinent to core themes will be developed. We will seek out and compare unique themes relevant to analytic subgroups (eg, age, gender, race, and ethnicity) to capture important thematic group differences. Final codes and memos will be compared and combined into overarching themes and subthemes. Themes will be discussed, refined, and named for the final analysis.

## Results

The study was funded by the National Institute on Drug Abuse on April 1, 2021. The trial is open and recruiting. We propose the enrollment of 60 caregivers by April 2025 and final data collection by September 2025. After final data analysis and writing of the results, manuscripts will be submitted to appropriate journals for dissemination. We anticipate submitting the main findings for publication by December 2025.

## Discussion

### Summary

This study is a protocol for an RCT testing an empirically derived mHealth app (RAISE) to support caregivers of detained adolescents in managing their stress and linking their adolescents with behavioral health treatment post release. The RAISE intervention includes mindfulness-based stress reduction techniques tailored specifically for caregivers of detained adolescents, informational resources for navigating the legal system and accessing mental health care, and self-monitoring and motivational messages. The RAISE intervention builds on prior research linking parenting stress and youth treatment engagement. Among caregivers of legally involved youth specifically, existing evidence suggests caregivers of youth court-mandated to treatment may be less likely to recognize their adolescent has a behavioral health problem [27] and that caregivers experiencing higher levels of their own mental health distress are more likely to recognize their child’s behavioral health needs (ie, youth problem recognition) [28]. Mindfulness-based stress reduction interventions have been shown to be effective for highly-stressed caregivers of adolescents [32,33], though investigations into the effects of addressing parenting stress among caregivers of detained youth on subsequent youth treatment engagement is limited. We anticipate that caregivers in the RAISE condition will report greater reductions in their parenting stress, as well as increased self-efficacy and motivation for their adolescent to engage in treatment (as mechanisms for youth behavioral health treatment engagement following release), compared to caregivers in the informational brochure condition.

Strengths of this study include the use of an RCT design to maximize internal validity and scientific rigor, the use of empirically supported intervention models (eg, mindfulness-based stress reduction), and the incorporation of mixed methods data collection and analysis to understand the intervention’s effectiveness. This study builds off prior participatory co-design work to create the RAISE app, which we anticipate will enhance intervention relevance and usability. A potential limitation is the reliance on self-report for primary outcomes, as we are unable to collect electronic health record data as collateral for service use due to the national recruitment approach. Furthermore, sample size may limit the current potential to analyze outcomes based on placement type and length of separation, which could be relevant to the intervention impact. We will descriptively explore these factors to inform a future larger trial.

### Conclusions

The current study aims to advance our understanding of how to support caregivers of detained adolescents in managing their parenting stress and the impact of doing so on their adolescents’ postrelease engagement with behavioral health services. Use of a mHealth intervention approach has the potential to increase scalability, and the current trial will provide insight into whether caregivers of detained adolescents find such an approach feasible and acceptable to use. Findings will inform a future large-scale hybrid trial and be used to provide empirically driven recommendations for systems related to supporting caregivers of detained adolescents.

## Appendix

Multimedia Appendix 1 SPIRIT (Standard Protocol Items: Recommendations for Interventional Trials) checklist.

Multimedia Appendix 2 CONSORT E-HEALTH checklist.

Multimedia Appendix 3 Caregiver consent form for the randomized controlled trial.

Multimedia Appendix 4 Caregiver exit interview guide.

Multimedia Appendix 5 Peer review report by the IPTA Interventions to Prevent and Treat Addictions Study Section (National Institutes of Health, USA).

## Abbreviations

mHealth: mobile health
RCT: randomized controlled trial

## Data Availability

Data sharing is not applicable to this study because no datasets have been generated or analyzed yet for the preparation of this protocol study. After analysis and dissemination, data generated and analyzed in the RCT will be available from the corresponding author upon reasonable request.
