# Supplementary material for: Stress Management Among Caregivers of Detained Youth: Protocol for Randomized Controlled Trial of the RAISE Web-Based mHealth App
Source: JMIR Res Protoc. 2025 Jul 10;14:e67511. doi: 10.2196/67511 (PMC12290427; doi:10.2196/67511)
Supplement: Multimedia Appendix 3 [file resprot_v14i1e67511_app3.pdf]

UNIVERSITY OF CALIFORNIA, SAN FRANCISCO
CONSENT TO PARTICIPATE IN A RESEARCH STUDY

Study Title: Reducing Parenting Stress to Facilitate Justice-Involved Youth's Treatment Engagement

Research Project Director:	Johanna Folk, PhD<br>Phone: 415-602-9521; E-mail: Johanna.Folk@ucsf.edu

Study Coordinator:	Elizabeth McBride<br>Phone: 415-713-3891; E-mail: Elizabeth.McBride@ucsf.edu

The purpose of this research study is to adapt and evaluate the efficacy of a mobile health intervention to reduce parenting stress among caregivers of detained youth. A research assistant from the University of California, San Francisco; Department of Psychiatry and Behavioral Sciences will explain the details of this study to you.

Research studies include only people who choose to take part. Please take your time to make your decision about participating and discuss your decision with your family or friends if you wish. If you have any questions, you may ask the researchers.

You are being asked to take part in this study because a child you care for has been involved in the juvenile justice system and placed in detention.

Why is this study being done?

The purpose of this study is to adapt and evaluate a parenting stress intervention to be delivered through mobile health technology for caregivers of justice-involved youth. We are asking caregivers to test out the intervention to see whether it is helpful for them.

This research is funded by the National Institute on Drug Abuse. We provide this funding disclosure so you can decide if this relationship affects your willingness to participate in this study.

How many people will take part in this study?

About 60 caregivers will participate in this part of the study.

What will happen if I take part in this research study?

You have completed a short screening that shows you are eligible to participate in the study. If you agree to participate, this is what will happen:

First, you will be randomly assigned (by a procedure similar to a coin flip) to receive either the mHealth parenting stress intervention or receive information describing ways to support one's adolescent during detention and community reentry via a brochure.

You will be asked to participate in 3 survey assessments. One at the beginning of your participation, another after 3 months, and a final survey after 6 months. Each survey will take about 50 minutes to complete. You will be paid $40 for each assessment you complete.

In addition to the $40 gift card for completing the baseline assessment, you will be entered into a drawing occurring at the end of the month for a $50 gift card if you complete your baseline assessment within 48 hours of receiving the link to access the baseline assessment.

At the 3-month follow up, you will be asked to participate in a qualitative interview. You will be paid $40 for participating in the interview. These interviews will be audio recorded. The recordings will only be used by the research staff and will not be shared with anyone in the court or legal system. To make sure your personal information is safe, the recordings will not have your name on them, and they will be stored on a secure UCSF online server.

If your youth is released into the community during the study, they will be eligible to participate in a qualitative interview as well. If you consent for your youth to participate, we will contact your youth and offer the opportunity for them to participate.

How long will I be in the study?

Participation in the study will last for 6 months.

Can I stop being in the study?

Yes. You can decide to stop at any time. Just tell the study researcher or staff person right away if you wish to stop being in the study. You are not required to participate and your decision to participate (or not) will have no impact on your own or your child's status in the justice, child welfare, or family court systems. If you decide not to participate or to quit the study, it will not affect any services you or your child might normally receive.

Also, the study researcher may stop you from taking part in this study at any time if he or she believes it is in your best interest, if there are concerns about the nature of your enrollment, if you do not follow the study rules, or if the study is stopped. If you are removed from the study, you will not continue to receive any study payments.

What side effects or risks can I expect from being in the study?

- The risks in this study are considered minimal.
- Some of the questions or group discussions may make you uncomfortable, but you are free to decline to answer any questions you do not wish to answer.
- For more information about risks, ask one of the researchers.

Are there benefits to taking part in the study?

You may not directly gain anything from participating in this study. However, the information you provide may help health professionals better understand and learn about how to reduce parenting stress among caregivers of detained youth through mobile health interventions.

What other choices do I have if I do not take part in this study?

You are free to choose not to participate in the study. If you decide not to take part in this study, there will be no penalty to you.

Will information about me be kept private?

We will do our best to make sure the personal information gathered for this study is kept private. However, we cannot guarantee total privacy. If you tell us you want to hurt yourself or someone else, or if someone is hurting you or someone else, we will get you help by telling the appropriate authorities. In a situation that we are worried about your safety, the research team may have to tell someone that you were a research participant in this study. If information from this study is published or presented at scientific meetings, your name and other personal information will not be used.

Researchers will use your information to conduct this study. Once the study is done using your information, we may share it with other researchers so they can use it for other studies in the future. We will not share your name or any other personal information that would let the researchers know who you are. We will not ask you for additional permission to share this de-identified information.

Organizations that may look at and/or copy your research records for research, quality assurance, and data analysis include representatives of the:

- Researcher and their support staff
- University of California
- National Institutes of Health
- Sponsor, the National Institute on Drug Abuse

This research is covered by a Certificate of Confidentiality from the National Institutes of Health. This means the researchers cannot release or use information, documents, or samples that may identify you in any action or suit unless you say it is okay. They also cannot provide them as evidence unless you have agreed. This protection includes federal, state, or local civil, criminal, administrative, legislative, or other proceedings. An example would be a court subpoena.

There are some important things that you need to know. The Certificate DOES NOT stop reporting that federal, state or local laws require. Some examples are laws that require reporting of child or elder abuse, some communicable diseases, and threats to harm yourself or others. The Certificate CANNOT BE USED to stop a sponsoring United States federal or state government agency from checking records or evaluating programs. The Certificate DOES NOT stop disclosures required by the federal Food and Drug Administration (FDA). The Certificate also DOES NOT prevent your information from being used for other research if allowed by federal regulations.

Researchers may release information about you when you say it is okay. For example, you may give them permission to release information to insurers, medical providers or any other persons not connected with the research. The Certificate of Confidentiality does not stop you from

willingly releasing information about your involvement in this research. It also does not prevent you from having access to your own information.

Are there any costs to me for taking part in this study?

No. You will not be charged for any of the study procedures.

Will I be paid for taking part in this study?

You will receive $40 for each assessment you complete and $40 for participating in the interview ($160 total for completing all three assessments and the interview).

In addition to the $40 gift card for completing the baseline assessment, you will be entered into a drawing occurring at the end of the month for a $50 gift card if you complete the baseline assessment within 48 hours of receiving the link to access the assessment questionnaire.

What are my rights if I take part in this study?

Taking part in this study is your choice. You may choose either to take part or not to take part in the study. If you decide to take part in this study, you may leave the study at any time. No matter what decision you make, there will be no penalty to you in any way. Signing this form does not take away any of your lawful rights.

Who can answer my questions about the study?

If you have any questions, concerns, or complaints about this study, you may contact the head researcher Dr. Johanna Folk at 415-602-9521.

If you wish to ask questions about the study or your rights as a research participant to someone other than the researchers or if you wish to voice any problems or concerns you may have about the study, please call the Institutional Review Board at 415-476-1814.

CONSENT

You have been given a copy of this consent form to keep.

PARTICIPATION IN RESEARCH IS VOLUNTARY. You have the right to decline to be in this study, or to withdraw from it at any point without penalty or loss of benefits to which you are otherwise entitled.

If you wish to participate in this study, you should sign below.

____________ ____

Date Caregiver/Legal Guardian Signature to Consent as a Participant

____________ ____

Date Caregiver/Legal Guardian Signature to Consent for Youth as a Participant

______________ ____
Date Person Obtaining Consent
