# Supplementary material for: Stress Management Among Caregivers of Detained Youth: Protocol for Randomized Controlled Trial of the RAISE Web-Based mHealth App
Source: JMIR Res Protoc. 2025 Jul 10;14:e67511. doi: 10.2196/67511 (PMC12290427; doi:10.2196/67511)
Supplement: Multimedia Appendix 4 [file resprot_v14i1e67511_app4.docx]

**Project RAISE Exit Interview (Caregiver)**

Interviewer: Thank you so much for taking the time to speak with us today. I would like to spend some time hearing more specifically about how things have been going since you started participating in Project RAISE. Before we start though, I want to remind you this interview is being audiotaped so I can make sure I remember everything you tell me accurately. These audio recordings will be erased once the content is transcribed and when it is transcribed, there will be nothing on the transcription that will allow someone to identify you. No one will hear those tapes except for myself and my research team. These audio recordings will never be made available to the court. I would like to remind you that everything you say here is confidential, with the exception of topics that relate to your own or your teen’s safety or the safety of others. So, whatever you say will not get back to your teen or the court unless it makes me concerned about someone’s safety. Do you have any questions before we get started?

Interviewer: I’d like to start off by just hearing about how things have been going for your family since you started participating in Project RAISE about 3 months ago. Tell me about what has been happening with you and your teen.

Interviewer: *(if teen is still in detention):* How have you been managing continuing to be separated from your teen?

Interviewer: *(if teen was released from detention):* Tell me about how the re-entry process went for your teen. How was it having them come home?

Interviewer: So, it sounds like things have been going ________. ***(If difficult, then indicate how challenging it can be to have a teen move back after being in detention (or to continue to be separated) and empathize with the caregiver about how upsetting and frustrating it can be to navigate challenges related to reunification/ongoing separation. If things have been going well, compliment the caregiver and build self-efficacy regarding their parenting and teen’s progress).***

Interviewer: How would you describe your stress over the last 3 months?

Interviewer: *(Focus on strengths/progress)* Tell me about something that has been going well for you as a caregiver in the last 3 months.

Interviewer: *(if teen was released from detention):* I’d like to start by understanding more about your teen’s participation in mental health or substance use treatment since they have been released into the community.

**[If not already completed as part of quantitative assessment: Administer CASA Questions (published assessment) Related to Teen’s Services Utilization; If completed, refer to quantitative assessment data]**

Interviewer: *(if teen participated in any services):* How easy or difficult was it to get your teen into care? What steps did you have to take in order to get them the help they needed?

Interviewer: *(if teen did not participate in any services):* When your teen was released, did you believe they needed any kind of mental health or substance use treatment? Did anyone else (like a probation officer) tell you your teen needed any kind of help? What got in the way of your teen being able to receive treatment?

***RAISE App Condition*:**

Interviewer: So, if you remember, the RAISE app had a lot of information about how to manage stress, including through practices like mindfulness. What I’d like to spend the rest of our time doing is understanding a little more specifically about how much or how little of the things you learned in the RAISE app have been useful to you.

Interviewer: Can you think of a time since you started using the RAISE app when you felt particularly stressed as a caregiver? ***(If yes, then continue).*** Tell me about what happened.

Interviewer: How easy or difficult was it for you to use some of the mindfulness and stress management practices you learned in the RAISE app in this situation?

***If they indicate they do not remember the strategies, ask:*** What do you remember learning from the program about mindfulness and managing stress?

***Follow-up as appropriate per response above:***

Interviewer: Which strategies did you use? What was easy about using that strategy in that situation? What was difficult about accessing those strategies in that situation?

Interviewer: ***(if a difficult interaction with teen related to communication)*** What did you discuss with your teen related to this conflict? How did the conversation go? How were you able to use mindfulness or full attention listening in this conversation? Or, what prevented you from using mindfulness or paying attention fully to your teen in this conversation?

Interviewer: How were the strategies you learned in the RAISE app helpful to you in managing being separated from your teen? In navigating the legal system? In their re-entry process? In getting them connected to treatment?

***Reactions to Project RAISE (both conditions):***

Interviewer: I would like to understand a bit more about your experiences with Project RAISE overall. Thinking back to when you first heard about Project RAISE. What were your first reactions when you heard about it?

Interviewer: What were your concerns about participating?

Interviewer: When you finally decided to participate, what made up your mind (or, what made you want to come)?

Interviewer: How did other people in your life - your husband/wife, boyfriend/girlfriend, friends - react when you told them you wanted to participate the program?

***RAISE app condition:***

Interviewer: Tell me about your experience with the web-based app. What made it easier to use? What made it more challenging? What did you like about using the app? What did you dislike? How did this app compare to others you have used?

Interviewer: If you were to tell other caregivers about the RAISE app, what would you tell them were your least favorite things or things you didn't like about it?

Interviewer: What would you tell them you liked the most about the app?

Interviewer: Would you recommend that other caregivers in similar situations use this app? Why or why not?

***Brochure condition:***

Interviewer: Tell me about your experience with the content covered in the brochure. How relevant was the content to you and your family? How helpful was it? What was one key thing you took away?

Interviewer: What information was missing from the brochure? What information was hard to understand?

Interviewer: How did the information in the brochure compare to resources you got from the juvenile justice system or other professionals your family is working with?

***Both conditions:***

Interviewer: Is there anything more you would like to share with me today about your experience with Project RAISE?

Interviewer: Thank you so much for participating in this interview today. Your responses are going to be very helpful for us as we continue to build resources for caregivers of teens in juvenile detention. It has been a pleasure to work with you and we wish you the best.
