# Supplementary material for: Stress Management Among Caregivers of Detained Youth: Protocol for Randomized Controlled Trial of the RAISE Web-Based mHealth App
Source: JMIR Res Protoc. 2025 Jul 10;14:e67511. doi: 10.2196/67511 (PMC12290427; doi:10.2196/67511)
Supplement: Multimedia Appendix 5 [file resprot_v14i1e67511_app5.pdf]

SUMMARY STATEMENT
(Privileged Communication)

PROGRAM CONTACT:
CARRIE Mulford
301-827-6473
carrie.mulford@nih.gov

Release Date: **06/17/2020**
Revised Date:

Application Number: **1 K23 DA050798-01A1**
Formerly: **1K23DA050798-01**

Principal Investigator

FOLK, JOHANNA BAILEY

Applicant Organization: UNIVERSITY OF CALIFORNIA, SAN FRANCISCO

Review Group: **IPTA**
Interventions to Prevent and Treat Addictions Study Section

Meeting Date: **06/04/2020**
Council: **OCT 2020**
Requested Start: **09/01/2020**
RFA/PA: **PA19-118**
PCC: **CM/CFM**

Project Title: **Reducing Parenting Stress to Facilitate Justice-Involved Youth's Treatment Engagement**
SRG Action: **Impact Score:20 Percentile:3 +**
Next Steps: **Visit https://grants.nih.gov/grants/next_steps.htm**
Human Subjects: 30-Human subjects involved - Certified, no SRG concerns
Animal Subjects: 10-No live vertebrate animals involved for competing appl.
Gender: 1A-Both genders, scientifically acceptable
Minority: 1A-Minorities and non-minorities, scientifically acceptable
Age: 1A-Children, Adults, Older Adults, scientifically acceptable

Project Year	Direct Costs Requested	Estimated Total Cost
1	186,850	201,798
2	186,850	201,798
3	186,850	201,798
4	186,850	201,798
5	186,850	201,798
TOTAL	934,250	1,008,990

ADMINISTRATIVE BUDGET NOTE: The budget shown is the requested budget and has not been adjusted to reflect any recommendations made by reviewers. If an award is planned, the costs will be calculated by Institute grants management staff based on the recommendations outlined below in the COMMITTEE BUDGET RECOMMENDATIONS section.

1K23DA050798-01A1 Folk, Johanna

RESUME AND SUMMARY OF DISCUSSION: This career development application requests support for the candidate to obtain training in substance use health services research, participatory research methods, and mHealth intervention evaluation, and to develop and test an mHealth mindfulness-based intervention to reduce caregiver stress in order to improve treatment engagement for justice-involved youth (JIY). During the discussion, reviewers were enthusiastic about the outstanding candidate with an impressive record of productivity and a well-aligned training plan and mentorship team. The research plan has the potential to address unmet treatment needs in JIY, the focus on participatory informatics and caregivers is innovative, and the mixed methods are rigorous. The candidate was responsive to the prior review, although concern remains that the assumption that reduced parenting stress will lead to improved youth engagement in treatment is not well supported and additional barriers to engagement are not addressed in the intervention. There is also still a lack of clarity about the added benefit of continuing to work with the same mentor. However, these remaining weaknesses did not detract from the panel’s high enthusiasm for the application, and there is a high likelihood that the candidate’s proposed career development and research plans will lead to a strong independent research career.

DESCRIPTION (provided by applicant): Justice-involved youth exhibit high rates of substance use and mental health symptoms, yet few receive treatment during detention or community re-entry. Once released into the community, caregivers must facilitate youth's treatment engagement, mobilizing significant resources and facing many barriers (e.g., transportation, mistrust) to do so. Parenting stress, which is heightened during youth detention and community reentry, is associated with greater perceived barriers to treatment, less youth therapeutic change throughout treatment, and premature treatment dropout. Addressing parenting stress improves youth treatment engagement and outcomes among youth exhibiting antisocial behavior, yet given the many barriers to treatment, novel approaches to intervention are needed; mobile health (mHealth) technology is one promising approach. Caregivers of justice-involved youth and system stakeholders are interested in mHealth treatment and mHealth addresses instrumental barriers (e.g., transportation) to treatment. Advances in technology and community engaged research allow for active stakeholder collaboration in mHealth application development, with no technological expertise required, through participatory informatics; caregiver involvement increases the likelihood the intervention will be relevant and efficacious. The purpose of this mixed-methods K23 study is to 1) develop a mHealth parenting stress intervention using participatory informatics; 2) assess the feasibility and acceptability of the participatory informatics approach and the intervention; 3) evaluate the intervention's preliminary efficacy in reducing parenting stress and increasing youth engagement in substance use or dual diagnosis treatment post-detention through a pilot RCT; and 4) understand systems-level factors that could influence eventual system adoption and sustainability. The overall goal of this K23 application is to provide protected, mentored time to expand Dr. Johanna Folk's (PI) capacities as an independent substance use health services researcher with expertise using participatory research methods to develop and evaluate novel interventions to engage justice-involved youth and families into youth substance use treatment. Dr. Folk will work with a team of experienced and knowledgeable mentors (Drs. Tolou-Shams, Aguilera, Knight, Arevian, and Chaplin) to increase her competency in: 1) substance use services research; 2) participatory research methods; and 3) mHealth methodology. The proposed research study is a logical extension of Dr. Folk's program of research to date which has spanned the developmental spectrum and focused on the complex interplay between justice involvement, substance use, mental health, and interpersonal relationships. This early career development award will provide the necessary candidate training and foundation for a larger R01 hybrid design clinical trial testing the efficacy of the mHealth parenting stress intervention designed during the K23, propelling a federally-funded program of

research designed to increase substance use treatment engagement and improve behavioral health outcomes for justice-involved youth and families.

PUBLIC HEALTH RELEVANCE: Parenting stress is a well-documented barrier to youth engagement in community-based substance use treatment. The proposed K23 project aims to develop and evaluate a mobile health parenting stress intervention for caregivers of justice-involved youth, a population with high rates of substance use and low rates of treatment engagement. The proposed research will expand the investigator's capacities as an independent health services investigator with expertise in: 1) substance use services research; 2) participatory methods; and 3) mHealth methodology.

CRITIQUE 1

Candidate: 1
Career Development Plan/Career Goals & Objectives: 1
Research Plan: 3
Mentor(s), Co-Mentor(s), Consultant(s), Collaborator(s): 1
Environment and Institutional Commitment to the Candidate: 1

Overall Impact: This K23 resubmission proposes training in substance use services research, participatory research methods, and mHealth methodology to promote Dr. Folk's career goal of becoming a health services researcher specializing in improving treatment access for justice-involved youth (JIY). The research plan proposes to develop and evaluate an mHealth intervention to reduce parenting stress for caregivers of JIY with the goal of increasing youth participation in substance use treatment. Formative work will include assessment of barriers and facilitators and intervention co-development with JIY caregivers, followed by a small open trial (n=13) to assess acceptability. A small randomized controlled study (n=60 caregivers) will be conducted to evaluate preliminary program impacts on parenting stress and youth treatment engagement, with stakeholder focus groups to explore systems-level factors related to sustainability. The candidate is outstanding with a productive publication record, the mentorship team is excellent with complementary expertise, and the training plan is well aligned with the candidate's career goals and research plan. The research plan addresses the significant public health issue of low treatment involvement for JIY youth with substance use problems, is innovative in its use of participatory informatics and focus on JIY caretakers, and is rigorously designed, including an iterative formative phase and randomized pilot trial with appropriate measures. A weakness with the proposed study concerns the limited scientific evidence that reducing parenting stress will substantially improve JIY treatment engagement. However, the strengths of the candidate and research plan outweigh this limitation.

1. Candidate:

Strengths

- Dr. Folk has been extremely productive, with 28 peer-reviewed pubs (57% first authored), 1 book chapter, and 2 NIDA grands (F31, NIH LRP)
- Her current T32 position at UCSF focuses on improving behavioral health treatment for justice-involved youth, extending her prior doctorial work with justice-involved populations
- Her career and training goals are clear and build logically on her current skill set and expertise

Weaknesses

- None noted by reviewer**.**

2. Career Development Plan/Career Goals & Objectives:

Strengths

- The career development plan includes 4 training goals relevant to Dr. Folk's proposed research project and long term career goals – to develop expertise in 1) substance use health services research, 2) participatory research methods and their application to mHealth intervention development, 3) mHealth intervention evaluation and analysis, and 4) professional development skills in manuscript writing, grant writing, and research partnerships
- The career development plan is clear and specific, with adequate didactic and mentored activities described for each training goal

Weaknesses

- None noted by reviewer.

3. Research Plan:

Strengths

- The intervention targets the low rates of behavioral health service use by JIY who need such services, a public health problem with long-term adverse impacts on JIY across the life span
- Preliminary data support positive impacts of Parenting Mindfully (PM) on parenting stress
- Innovative aspects include the use of participatory informatics, targeting JIY caregivers for intervention, and use of mHealth in this context
- The participatory co-development process for Aim 1 is thoughtfully planned with multiple iterations to facilitate meaningful stakeholder input
- The pilot includes a randomized design, 3- and 6-month assessments, measures consistent with the conceptual model, and analysis of feasibility, acceptability, primary and secondary outcomes, and use metrics

Weaknesses

- While parenting stress has been shown to be associated with greater perceived barriers to youth treatment, scientific rationale is limited to support assumption that reduced parenting stress will lead to improved youth engagement in treatment
- Financial and logistical barriers to youth treatment are not being addressed in the intervention and will likely contribute significantly to youth engagement in treatment

4. Mentor(s), Co Mentor(s), Consultant(s), Collaborator(s):

Strengths

- The mentorship team includes Dr. Tolou-Shams as primary mentor, Drs. Aguilera, Knight, and Arevian as co-mentors, and Dr. Chaplin as an off-site consultant – all experienced mentors with relevant expertise in JIY services, mHealth technology and analyses, substance use services and family factors related to engagement, participatory informatics, and adolescent substance use respectively

- The response to reviewers makes a persuasive case for Dr. Tolou-Shams role as primary mentor for the K award, despite the current mentorship role she plays in Dr. Folk's T32. Her expertise is best suited to that role, and the two have a productive working relationship.

Weaknesses

- None noted by reviewer.

5. Environment and Institutional Commitment to the Candidate:

Strengths

- The environment at UCSF is excellent for supporting the proposed K award
- The institution shows a strong commitment to the candidate and intends to transition her to a faculty position in winter 2020 with 100% time to devote to the K award research and training

Weaknesses

- None noted by reviewer.

Study Timeline:

Strengths

- None noted by reviewer.

Weaknesses

- None noted by reviewer.

Protections for Human Subjects:

Acceptable Risks and Adequate Protections

- Human subjects' protections are acceptable

Data and Safety Monitoring Plan (Applicable for Clinical Trials Only):

Acceptable

- DSMP is acceptable.

Inclusion Plans:

- Sex/Gender: Distribution justified scientifically
- Race/Ethnicity: Distribution justified scientifically
- For NIH-Defined Phase III trials, Plans for valid design and analysis: Not applicable
- Inclusion/Exclusion Based on Age: Distribution justified scientifically
- The randomized pilot trial will enroll male and female adolescents aged 12-17. Approximately 75% are estimated to be male and 27% are estimated to be Latinx, 59% African American, 3% White, 8% Asian or Pacific Islander, 27% multi-ethnic, and 3% other. Male and female caretakers (approximately 75% female) and male and female stakeholders (approximately 64% female) will also be enrolled, including individuals of diverse racial/ethnic backgrounds.

Vertebrate Animals:

Not Applicable (No Vertebrate Animals)

Biohazards:

Not Applicable (No Biohazards)

Resubmission:

- The application was responsive to prior concerns, including clarification of the conceptual model, discussion of the developmental range of the JIY, and reduction in the length of the assessment battery.

Training in the Responsible Conduct of Research:

Acceptable

Comments on Format (Required):

- Recorded lecture plus readings, exercises, and online case discussions; in person seminars and works in progress sessions; CITI course

Comments on Subject Matter (Required):

- Research ethics, conflict of interest, mentor/mentee responsibilities, collaborative research, peer review, and others

Comments on Faculty Participation (Required; not applicable for mid- and senior-career awards):

- Experienced faculty will lead coursework and seminars and WIPS

Comments on Duration (Required):

- 3-hour course repeated every 4 years plus 2.75 hours weekly in year 1, biweekly in subsequent years

Comments on Frequency (Required):

- 30-hour course repeated every 4 years plus 2.75 hours weekly in year 1, biweekly in subsequent years

Resource Sharing Plans:

Acceptable

Budget and Period of Support:

Recommend as Requested

CRITIQUE 2

Candidate: 1
Career Development Plan/Career Goals & Objectives: 1
Research Plan: 2
Mentor(s), Co-Mentor(s), Consultant(s), Collaborator(s): 2
Environment and Institutional Commitment to the Candidate: 1

Overall Impact: This K23 application proposes training and research plans to enhance Dr. Folk's skills in health services research, participatory methods, mHealth interventions, and parent-focused interventions. Dr. Folk is an accomplished early career researcher with an impressive number of publications and previous NIDA funding. She has assembled a team of researchers with experience in the identified training areas, considerable records of mentorship of early career researchers, and a clear commitment to her development. The institutional environment has supports in place for early career researchers, particularly through the Clinical and Translational Science Institute. The research plan focuses on reducing parenting stress for caregivers of justice involved youth, an important underserved population, through the co-development of an mHealth intervention. The application is very responsive to prior feedback, with two exceptions. The proposal did not clearly explain what could be gained by continuing to work with the same mentor that was not available during the T32. In terms of the research plan, it remains unclear whether this intervention could stand alone and improve linkage to care for JIY, however, the use of participatory methods, which include families, probation officers, and behavioral health providers, is nonetheless likely to uncover other potential barriers that can be addressed with additional program content. Consequently, despite these two limitations, enthusiasm remains high.

1. Candidate:

Strengths

- The candidate is a clinical psychologist with evidence of high productivity, including 28 peer-reviewed publications and two NIDA funded grants.
- Her focus is on increasing substance use treatment engagement and improving behavioral health outcomes for justice involved youth (JIY), an underserved population. The training plan is clearly linked with her long-term goal of obtaining a faculty position in an academic medical center where she can conduct health services research related to applied interventions for JIY and families.

Weaknesses

- None noted by reviewer.

2. Career Development Plan/Career Goals & Objectives:

Strengths

- Training goals are relevant to the candidate's proposed career path. Planned coursework and mentorship experiences are in alignment with each goal. (major)

Weaknesses

- None noted by reviewer.

3. Research Plan:

Strengths

- Research focuses on a population at risk for substance abuse and limited treatment engagement. Candidate has clarified conceptual theory about how parenting stress is linked to adolescent substance use and engagement in care. (major)
- Parents of JIY and experience unique risk factors including stigma and mistrust of the justice system that should be addressed in interventions for this population.

- mHealth interventions may improve reach, feasibility, and acceptability, particularly when developed in partnership with users. The proposed application will make use of participatory informatics to develop and evaluate an mHealth intervention. (major)
- Content from the Parenting Mindfully intervention will be used as a resource for the development of the intervention, which will increase the feasibility of the project. The candidate has clarified that if the participatory methods point to the need for other content, that content will be identified by the research team. (major)
- Candidate has addressed how the intervention may be tailored for developmental appropriateness for the range of ages targeted by the intervention. (major)
- The quantitative and qualitative methods described in each phase appear appropriate (major)
- The length of the battery has been reduced in response to reviewer concerns (moderate)

Weaknesses

- The proposal does read as a little prescriptive for a participatory study (e.g., states that caregivers will use the app for 3 months), however, it is useful to have a starting point that can be adapted as relevant. The proposal notes a backup plan for if the PM intervention does not meet the needs identified by the participants. (moderate)
- The notion that reducing parenting stress will be sufficient to engage youth in care is still not completely satisfying. It may be the case that human involvement is needed to link JIY in care, and that this app could function as a companion to that process. However, these issues are likely to come out in the study, particularly in Aim 3. (moderate)
- The proposal mentions unique risk factors, such as stigma, but does not mention how the program will address these factors. (moderate)

4. Mentor(s), Co Mentor(s), Consultant(s), Collaborator(s):

Strengths

- Dr. Tolou-Shams has a history of mentorship with 3 early career K awards, 1 NIDA diversity supplement, 3 NIH LRPs, 2 T32s, and an R25 for mentoring underrepresented minority trainees. She will provide general mentoring and expertise related to family-based interventions and ethical considerations for underserved populations. (major)
- The assembled team has relevant expertise for the training goals and research plan (major)
- Dr. Aguilera provides expertise on technology-based interventions and longitudinal data analysis.
- Dr. Knight provides expertise on substance use services research
- Dr. Arevian provides expertise in participatory approaches to mHealth development, including qualitative coding methods
- Dr. Chapin is the developer of Parenting Mindfully, and will serve as a consultant to provide guidance on the adaptation of the model
- The proposal outlines how mentorship will occur with mentors at other sites (major)

Weaknesses

- Candidate was moderately responsive to concerns from the previous review about the continuation with the same mentor from the T32, and what new training opportunities can be gained relative to what was previously available (moderate)

5. Environment and Institutional Commitment to the Candidate:

Strengths

- Department of Psychiatry will transition Dr. Folk to a faculty position with 100% protected time to engage in research and training
- UCSF's Clinical and Translational Science Institute (CTSI) can provide a network of future collaborators, in addition to training opportunities
- Letters of support are highly positive and indicate confidence in potential for success

Weaknesses

- None noted by reviewer.

Study Timeline:

Strengths

- The timeline is clearly presented and appears feasible.

Weaknesses

- None noted by reviewer.

Protections for Human Subjects:

Acceptable Risks and Adequate Protections

- Risks are limited to subjective discomfort and breach of confidentiality. Strategies are in place to mitigate the likelihood of these risks

Data and Safety Monitoring Plan (Applicable for Clinical Trials Only):

Acceptable

- The PI will be responsible for responding to AEs or SAEs and reporting to the IRB, the mentoring team, DSMB, and NIH. The DSMB will be formed before the trial begins and will include researchers with experience with clinical trials, mental health research with underserved populations, and research with youth and families. A quantitative methodologist is also recommended.

Inclusion Plans:

- Sex/Gender: Distribution justified scientifically
- Race/Ethnicity: Distribution justified scientifically
- For NIH-Defined Phase III trials, Plans for valid design and analysis: Not applicable
- Inclusion/Exclusion Based on Age: Distribution justified scientifically
- No participants will be excluded on the basis of sex, race, or ethnicity. The intervention is designed to improve outcomes for adolescents in juvenile detention (12-17 years) by intervening with parents (18 years or older, based on need to consent)

Vertebrate Animals:

Not Applicable (No Vertebrate Animals)

Biohazards:
Not Applicable (No Biohazards)

Resubmission:

Training in the Responsible Conduct of Research:
Acceptable
Comments on Format (Required):

- formal online and face to face coursework through CTSI; CITI training

Comments on Subject Matter (Required):

- research ethics with human subjects, conflict of interest, mentor and mentee responsibilities, collaborative research, peer review, data acquisition, research misconduct, responsible authorship and the scientist as a responsible member of society, and ethics specific to proposed research, including adolescent substance use, recruitment of vulnerable populations, and mobile health technology

Comments on Faculty Participation (Required; not applicable for mid- and senior-career awards):

- Barbara Koenig PhD, Professor of Health and Aging in the School of Nursing and Winston Chiong MD, PhD, Assistant Professor of Neurology in the School of Medicine; Recorded content by Dr. Bernard Lo, Professor of Medicine Emeritus at UCSF, Director of the Greenwall Foundation and an international expert on research ethics and Dr. Deborah Grady, Professor of Medicine and Director of the CTSI Training Programs; mentorship team

Comments on Duration (Required):

- Epi 201 includes 8 hours of lecture recordings and students spend approximately 30 hours completing readings, viewing lectures, and participating in interactive case discussions. CTSI K Scholar Program seminars (1 hour) and WIPS (1.75 hours) occur weekly in the scholar's first year, biweekly in subsequent years

Comments on Frequency (Required):

- Responsible Conduct of Research course in years 1 and 5; CITI module every three years; K Program seminars, WIPS, and mentor training will occur for the duration of the career development award

Resource Sharing Plans:
Acceptable

Budget and Period of Support:
Recommend as Requested

CRITIQUE 3

Candidate: 1
Career Development Plan/Career Goals & Objectives: 1
Research Plan: 3
Mentor(s), Co-Mentor(s), Consultant(s), Collaborator(s): 1
Environment and Institutional Commitment to the Candidate: 1

Overall Impact: This is a resubmission of K23 application from an outstanding candidate who is surrounded by a supportive and capable mentorship team. The career development plan is strong and specific, and will build Dr. Folk's expertise in several interlocking areas (substance use health services research, participatory methods in mHealth intervention development, statistical analysis of outcomes, and professional development) to advance her research in parenting stress for adolescents involved in the juvenile justice system. This is a highly productive candidate with a strong emerging recording of scientific contributions. The underlying intervention development strategy of participatory informatics is novel, particularly for this population of justice-involved youth and their caregivers. The proposed research study is rigorous, with considerable attention to detail. Some concerns remain about the ultimate impact of parental stress reduction on adolescent substance use, however on balance they do not detract from the strengths of the candidate or the application. There is a high likelihood that the candidate will use the K23 experience to gain the skills necessary to ultimately progress to an independent investigator. Thus, enthusiasm for this application is high.

1. Candidate:

Strengths

- Highly productive new, early stage scientist with an impressive record of scholarship considering her career stage.
- Currently a postdoctoral fellow preparing to transition to a faculty appointment, has been funded on T32 and NRSA awards.
- Already has experience in proposal development and grant writing (as attested by mentor letters).
- The candidate has well-grounded expertise in issues of adolescent substance use and correctional healthcare for adults.

Weaknesses

- None noted

2. Career Development Plan/Career Goals & Objectives:

Strengths

- The career development plan is detailed and well-described, matching training activities to the main focal areas of substance use services research, participatory informatics, mHealth, and data analysis.
- Plan includes formal coursework and targeted mentorship. Mentor expertise and commitment are well-aligned with training goals.

Weaknesses

- None noted

3. Research Plan:

Strengths

- Youth involved in the juvenile justice system are at high risk for poor outcomes. Parents of youth returning from detention face multiple stressors and there is a need for interventions that can help to mitigate the adverse effects of stress on parents and youth outcomes.
- Good overview of the theoretical basis and literature supporting the link between parental stress and youth engagement in risky behaviors.
- Detailed development/adaptation plan for the intervention.
- Small RCT design with attention to rigor; if successful the approach could yield strong preliminary data to support future scientific efforts.

Weaknesses

- A good case is made that parenting stress is associated with adverse youth outcomes. Nevertheless, as a target of intervention parental stress may be far removed from youth outcomes, especially to the extent that this relationship is entangled with other parenting and parent-youth relationship variables that are less amenable to change.
- Youth outcomes are limited to treatment engagement ascertained via record review. This may be a missed opportunity to capture youth outcomes and perspectives directly to develop more compelling preliminary data for a future R01 application.

4. Mentor(s), Co Mentor(s), Consultant(s), Collaborator(s):

Strengths

- Excellent team of mentors with extensive experience to guide the candidate’s career development.
- Chief Mentor Tolou-Shams is a senior investigator who already has a productive relationship with the candidate and brings significant expertise in the intersection of family interventions, juvenile justice, and mHealth.
- Other mentors complement the team and lend specific expertise in research-driven mHealth intervention development (Aguilera, Arevian), implementation science in the juvenile justice arena (Knight), and the Parenting Mindfully intervention (Chaplin).

Weaknesses

- None noted.

5. Environment and Institutional Commitment to the Candidate:

Strengths

- Very strong letters of support that speak to the exceptional promise of the candidate.
- Evidence of institutional commitment, including 100% protected time with K23, and plans to transition the candidate from her current postdoc to a faculty position later this year.

Weaknesses

- None noted.

Study Timeline:

Strengths

- The study timeline is well-described and reasonable.

Weaknesses

- None noted.

Protections for Human Subjects:

Acceptable Risks and Adequate Protections

- No concerns

Data and Safety Monitoring Plan (Applicable for Clinical Trials Only):

Acceptable

- No concerns; there will be a DSMB.

Inclusion Plans:

- Sex/Gender: Distribution justified scientifically
- Race/Ethnicity: Distribution justified scientifically
- For NIH-Defined Phase III trials, Plans for valid design and analysis: Not applicable
- Inclusion/Exclusion Based on Age: Distribution justified scientifically
- Study will recruit 60 dyads of justice-involved youth and their caregivers, as well as system stakeholders. Youth participation will be limited to record review. No exclusions based on sex/gender or race/ethnicity. It is expected that 75% of youth will be male; while 75% of caregivers will be female and 64% of justice system stakeholders will be female.

Vertebrate Animals:

Not Applicable (No Vertebrate Animals)

Biohazards:

Not Applicable (No Biohazards)

Resubmission:

- The application was responsive to prior reviewer comments.

Training in the Responsible Conduct of Research:

Acceptable

Comments on Format (Required):

- Coursework at UCSF, online training via CITI, K scholar program seminars and works-in-progress sessions

Comments on Subject Matter (Required):

- Ethics in conduct of human subjects research, conflicts of interest, mentor/mentee roles, collaboration, etc.

Comments on Faculty Participation (Required; not applicable for mid- and senior-career awards):

- UCSF Faculty teach the courses (Dr. Lo, Dr. Grady, Dr. Koenig, Dr Chiong).

Comments on Duration (Required):

- Epidemiology course is 8 hours of lecture, 30 hours of reading. Seminars and works-in-progress session aggregate to ~3 hours weekly in year 1, biweekly thereafter

Comments on Frequency (Required):

- Responsible conduct of research course bookends the K23 experience in years 1 and 5. Seminars and ongoing mentorship activities occur throughout the award.

Resource Sharing Plans:

Not Applicable (No Relevant Resources)

Budget and Period of Support:

Recommend as Requested

THE FOLLOWING SECTIONS WERE PREPARED BY THE SCIENTIFIC REVIEW OFFICER TO SUMMARIZE THE OUTCOME OF DISCUSSIONS OF THE REVIEW COMMITTEE, OR REVIEWERS' WRITTEN CRITIQUES, ON THE FOLLOWING ISSUES:

PROTECTION OF HUMAN SUBJECTS: ACCEPTABLE

INCLUSION OF WOMEN PLAN: ACCEPTABLE

INCLUSION OF MINORITIES PLAN: ACCEPTABLE

INCLUSION ACROSS THE LIFESPAN: ACCEPTABLE

COMMITTEE BUDGET RECOMMENDATIONS: The budget was recommended as requested.

Footnotes for 1 K23 DA050798-01A1; PI Name: Folk, Johanna Bailey

+ Derived from the range of percentile values calculated for the study section that reviewed this application.

NIH has modified its policy regarding the receipt of resubmissions (amended applications).See Guide Notice NOT-OD-18-197 at https://grants.nih.gov/grants/guide/notice-files/NOT-OD-18-197.html. The impact/priority score is calculated after discussion of an application by averaging the overall scores (1-9) given by all voting reviewers on the committee and multiplying by 10. The criterion scores are submitted prior to the meeting by the individual reviewers assigned to an application, and are not discussed specifically at the review meeting or calculated into the overall impact score. Some applications also receive a percentile ranking. For details on the review process, see http://grants.nih.gov/grants/peer_review_process.htm#scoring.

MEETING ROSTER

Interventions to Prevent and Treat Addictions Study Section
Risk, Prevention and Health Behavior Integrated Review Group
CENTER FOR SCIENTIFIC REVIEW
IPTA
06/04/2020 - 06/05/2020

Notice of NIH Policy to All Applicants: Meeting rosters are provided for information purposes only. Applicant investigators and institutional officials must not communicate directly with study section members about an application before or after the review. Failure to observe this policy will create a serious breach of integrity in the peer review process, and may lead to actions outlined in NOT-OD-14-073 at https://grants.nih.gov/grants/guide/notice-files/NOT-OD-14-073.html and NOT-OD-15-106 at https://grants.nih.gov/grants/guide/notice-files/NOT-OD-15-106.html, including removal of the application from immediate review.

CHAIRPERSON(S)

WALTON, MAUREEN A, PHD, MPH
PROFESSOR
DEPARTMENT OF PSYCHIATRY
UNIVERSITY OF MICHIGAN
ANN ARBOR, MI 48109

MEMBERS

ALESSI, SHEILA MARIE, PHD *
ASSOCIATE PROFESSOR
DEPARTMENT OF PSYCHIATRY
CALHOUN CARDIOLOGY CENTER
UNIVERSITY OF CONNECTICUT HEALTH CENTER
FARMINGTON 06030

BERKEL, CADY, PHD *
ASSOCIATE RESEARCH PROFESSOR
REACH INSTITUTE
SOUTHWEST INTERDISCIPLINARY RESEARCH CENTER
ARIZONA STATE UNIVERSITY
TEMPE, AZ 85284

BERMAN, MITCHELL E, PHD *
PROFESSOR AND DEPARTMENT HEAD
DEPARTMENT OF PSYCHOLOGY
MISSISSIPPI STATE UNIVERSITY
MISSISSIPPI STATE, MS 39762

BERNSTEIN, STEVEN L, MD
PROFESSOR AND VICE CHAIR
DEPARTMENT OF EMERGENCY MEDICINE
YALE UNIVERSITY SCHOOL OF MEDICINE
NEW HAVEN, CT 06519

BRADIZZA, CLARA M, PHD
PROFESSOR
SCHOOL OF SOCIAL WORK
UNIVERSITY AT BUFFALO
STATE UNIVERSITY OF NEW YORK
BUFFALO, NY 14203

BUCKNER, JULIA D, PHD *
PROFESSOR AND DIRECTOR OF CLINICAL TRAINING
DEPARTMENT OF PSYCHOLOGY
LOUISIANA STATE UNIVERSITY
BATON ROUGE, LA 70803

COLEMAN-COWGER, VICTORIA HOPE, PHD
CLINICAL RESEARCH DIRECTOR
THE EMMES CORPORATION
ROCKVILLE, MD 20850

CROPSEY, KAREN L, PSYD
PROFESSOR
DEPARTMENT OF PSYCHIATRY AND BEHAVIORAL NEUROLOGY
UNIVERSITY OF ALABAMA AT BIRMINGHAM
BIRMINGHAM, AL 35294

DUNN, KELLY E., PHD *
ASSOCIATE PROFESSOR
DEPARTMENT OF PSYCHIATRY AND BEHAVIORAL SCIENCES
SCHOOL OF MEDICINE
JOHNS HOPKINS UNIVERSITY
BALTIMORE, MD 21224-6823

FRIEDMANN, PETER D, MD, MPH
PROFESSOR
DEPARTMENT OF MEDICINE
UNIVERSITY OF MASSACHUSETTS
MEDICAL SCHOOL - BAYSTATE
SPRINGFIELD, MA 01107

GRANT, SEAN, PHD *
ASSISTANT PROFESSOR
RICHARD M. FAIRBANKS SCHOOL OF PUBLIC HEALTH
INDIANA UNIV-PURDUE UNIV AT INDIANAPOLIS
INDIANAPOLIS, IN 46202

GRYCZYNSKI, JAN, PHD
SENIOR RESEARCH SCIENTIST
FRIENDS RESEARCH INSTITUTE
BALTIMORE, MD 21201

HETTEMA, JENNIFER ELIN, PHD *
ASSOCIATE PROFESSOR
DEPARTMENT OF FAMILY
AND COMMUNITY MEDICINE
UNIVERSITY OF NEW MEXICO SCHOOL OF MEDICINE
ALBUQUERQUE 87131

HILL, KEVIN P., MD *
ASSOCIATE PROFESSOR OF PSYCHIATRY
HARVARD MEDICAL SCHOOL
BOSTON, MA 02215

HITSMAN, BRIAN L, PHD
ASSOCIATE PROFESSOR
DEPARTMENT OF PREVENTIVE MEDICINE
FEINBERG SCHOOL OF MEDICINE
NORTHWESTERN UNIVERSITY
CHICAGO, IL 60611

INGERSOLL, KAREN S, PHD *
PROFESSOR
DEPARTMENT OF PSYCHIATRY
AND NEUROBEHAVIORAL SCIENCES
UNIVERSITY OF VIRGINIA
CHARLOTTESVILLE, VA 22911

KAHLER, CHRISTOPHER W., PHD *
PROFESSOR AND CHAIR
DEPARTMENT OF BEHAVIORAL
AND SOCIAL SCIENCES
CENTER FOR ALCOHOL AND ADDICTION STUDIES
BROWN UNIVERSITY SCHOOL OF PUBLIC HEALTH
PROVIDENCE, RI 02912

KIM-MOZELESKI, JIN E., PHD *
ASSISTANT PROFESSOR
DEPARTMENT OF POPULATION AND
QUANTITATIVE HEALTH SCIENCES
CASE WESTERN RESERVE UNIVERSITY
CLEVELAND, OH 44106

LARSON, MARY JO, PHD
SENIOR SCIENTIST
INSTITUTE FOR BEHAVIORAL HEALTH
HELLER SCHOOL FOR SOCIAL POLICY AND MANAGEMENT
BRANDEIS UNIVERSITY
WALTHAM, MA 02454

LITT, DANA M, PHD *
ASSOCIATE PROFESSOR
DEPARTMENT OF HEALTH BEHAVIOR AND HEALTH
SYSTEMS
SCHOOL OF PUBLIC HEALTH SYSTEMS
UNIVERSITY OF NORTH TEXAS
FORT WORTH 76107

LOVEJOY, TRAVIS IAN, MPH, PHD *
ASSOCIATE PROFESSOR
DEPARTMENT OF PSYCHIATRY
SCHOOL OF MEDICINE
OREGON HEALTH AND SCIENCE UNIVERSITY
PORTLAND, OR 97239

MAHABEE-GITTENS, E. MELINDA, MD
PROFESSOR
DIVISION OF EMERGENCY MEDICINE
CINCINNATI CHILDREN'S HOSPITAL MEDICAL CENTER
CINCINNATI, OH 45229

MAISTO, STEPHEN A, PHD *
PROFESSOR
DEPARTMENT OF PSYCHOLOGY
SYRACUSE UNIVERSITY
SYRACUSE, NY 13244

MCGOVERN, MARK P, PHD
PROFESSOR
DEPARTMENT OF PSYCHIATRY
AND BEHAVIORAL SCIENCES
STANFORD UNIVERSITY
PALO ALTO, CA 93404

MENDELSON, TAMAR, PHD
PROFESSOR
DEPARTMENT OF MENTAL HEALTH
JOHNS HOPKINS BLOOMBERG SCHOOL OF PUBLIC HEALTH
BALTIMORE, MD 21205

MILLER, MARY ELIZABETH, PHD *
ASSISTANT PROFESSOR
DEPARTMENT OF PSYCHIATRY
UNIVERSITY OF MISSOURI
COLUMBIA, MO 65211

MONTGOMERY, LATRICE, PHD *
ASSISTANT PROFESSOR
ADDICTION SCIENCES DIVISION
DEPARTMENT OF PSYCHIATRY
AND BEHAVIORAL NEUROSCIENCE
UNIVERSITY OF CINCINNATI COLLEGE OF MEDICINE
CINCINNATI, OH 45229

NOONAN, DEVON, PHD *
ASSOCIATE PROFESSOR
SCHOOL OF NURSING
DUKE UNIVERSITY
DURHAM, NC 27710

OLIVETO, ALISON, PHD
PROFESSOR AND VICE CHAIR FOR RESEARCH
DEPARTMENT OF PSYCHIATRY
UNIVERSITY OF ARKANSAS FOR MEDICAL SCIENCES
LITTLE ROCK, AR 72205

OSILLA, KAREN C, PHD
SENIOR BEHAVIORAL SCIENTIST
RAND CORPORATION
SANTA MONICA, CA 90404

PARROTT, DOMINIC, PHD *
PROFESSOR
DEPARTMENT OF PSYCHOLOGY
GEORGIA STATE UNIVERSITY
ATLANTA, GA 30302

PIPER, MEGAN E, PHD
ASSOCIATE PROFESSOR
DEPARTMENT OF MEDICINE
UNIVERSITY OF WISCONSIN-MADISON
MADISON, WI 53711

SCHEUERMANN, TANEISHA SHANI, PHD *
ASSISTANT PROFESSOR
DEPARTMENT OF PREVENTIVE MEDICINE AND
PUBLIC HEALTH
UNIVERSITY OF KANSAS MEDICAL CENTER
KANSAS CITY, KS 66160

STEIN, MICHAEL D, MD
PROFESSOR AND CHAIR
DEPARTMENT OF HEALTH LAW, POLICY AND MANAGEMENT
BOSTON UNIVERSITY SCHOOL OF PUBLIC HEALTH
BOSTON, MA 02118

STEINBERG, MARC L, PHD *
ASSOCIATE PROFESSOR
DIVISION OF ADDICTION PSYCHIATRY
ROBERT WOOD JOHNSON MEDICAL SCHOOL
RUTGERS, THE STATE UNIVERSITY OF NEW JERSEY
NEW BRUNSWICK, NJ 08901

TINDLE, HILARY A, MD, MPH
ASSOCIATE PROFESSOR
DIVISION OF INTERNAL MEDICINE AND PUBLIC HEALTH
VANDERBILT UNIVERSITY MEDICAL CENTER
NASHVILLE, TN 37203

TSOH, JANICE Y, PHD
PROFESSOR
DEPARTMENT OF PSYCHIATRY
LANGLEY PORTER PSYCHIATRIC INSTITUTE
UNIVERSITY OF CALIFORNIA SAN FRANCISCO
SAN FRANCISCO, CA 94143

TSUI, JUDITH, MD, MPH *
ASSOCIATE PROFESSOR OF MEDICINE
DIVISION OF GENERAL INTERNAL MEDICINE
UNIVERSITY OF WASHINGTON SCHOOL OF MEDICINE
HARBORVIEW MEDICAL CENTER
SEATTLE, WA 98122

VELASQUEZ, MARY M, PHD
CENTENNIAL PROFESSOR AND DIRECTOR
HEALTH BEHAVIOR RESEARCH
AND TRAINING INSTITUTE
STEVE HICKS SCHOOL OF SOCIAL WORK
UNIVERSITY OF TEXAS AT AUSTIN
AUSTIN, TX 78712

VINCI, CHRISTINE, PHD *
ASSISTANT MEMBER
DEPARTMENT OF HEALTH OUTCOMES AND BEHAVIOR
MOFFITT CANCER CENTER
TAMPA, FL 33612

WINSTANLEY, ERIN L, PHD *
ASSOCIATE PROFESSOR
DEPARTMENT OF BEHAVIORAL MEDICINE AND PSYCHIATRY
WEST VIRGINIA UNIVERSITY, SCHOOL OF MEDICINE
MORGANTOWN, WV 26506

YI, RICHARD, PHD *
PROFESSOR
DEPARTMENT OF PSYCHOLOGY
COFRIN LOGAN CENTER FOR ADDICTION RESEARCH AND
TREATMENT
UNIVERSITY OF KANSAS
LAWRENCE, KS 66045

SCIENTIFIC REVIEW OFFICER

MINTZER, MIRIAM, PHD
SCIENTIFIC REVIEW OFFICER
CENTER FOR SCIENTIFIC REVIEW
NATIONAL INSTITUTES OF HEALTH
BETHESDA, MD 20892

EXTRAMURAL SUPPORT ASSISTANT

FAYEMIWO, TOLU, MS
EXTRAMURAL SUPPORT ASSISTANT
CENTER FOR SCIENTIFIC REVIIEW
NATIONAL INSTITUTES OF HEALTH
BETHESDA, MD 20892

* Temporary Member. For grant applications, temporary members may participate in the entire meeting or may review only selected applications as needed.

Consultants are required to absent themselves from the room during the review of any application if their presence would constitute or appear to constitute a conflict of interest.
